# Enhancing Lung Ultrasound Diagnostics: A Clinical Study on an Artificial Intelligence Tool for the Detection and Quantification of A-Lines and B-Lines

**DOI:** 10.3390/diagnostics14222526

**Published:** 2024-11-12

**Authors:** Mahdiar Nekoui, Seyed Ehsan Seyed Bolouri, Amir Forouzandeh, Masood Dehghan, Dornoosh Zonoobi, Jacob L. Jaremko, Brian Buchanan, Arun Nagdev, Jeevesh Kapur

**Affiliations:** 1 Exo Imaging, Santa Clara, CA 95054, USA; 2 Department of Radiology & Diagnostic Imaging, University of Alberta, Edmonton, AB T6G 2R3, Canada; 3 Department of Critical Care Medicine, University of Alberta, Edmonton, AB T6G 2R3, Canada; 4 Alameda Health System, Highland General Hospital, University of California San Francisco, San Francisco, CA 94143, USA; 5 Department of Diagnostic Imaging, National University of Singapore, Singapore 119228, Singapore

**Keywords:** A-lines, B-lines, lung ultrasound, machine learning, artificial intelligence

## Abstract

**Background/Objective:** A-lines and B-lines are key ultrasound markers that differentiate normal from abnormal lung conditions. A-lines are horizontal lines usually seen in normal aerated lungs, while B-lines are linear vertical artifacts associated with lung abnormalities such as pulmonary edema, infection, and COVID-19, where a higher number of B-lines indicates more severe pathology. This paper aimed to evaluate the effectiveness of a newly released lung ultrasound AI tool (ExoLungAI) in the detection of A-lines and quantification/detection of B-lines to help clinicians in assessing pulmonary conditions. **Methods**: The algorithm is evaluated on 692 lung ultrasound scans collected from 48 patients (65% males, aged: 55 ± 12.9) following their admission to an Intensive Care Unit (ICU) for COVID-19 symptoms, including respiratory failure, pneumonia, and other complications. **Results**: ExoLungAI achieved a sensitivity of 91% and specificity of 81% for A-line detection. For B-line detection, it attained a sensitivity of 84% and specificity of 86%. In quantifying B-lines, the algorithm achieved a weighted kappa score of 0.77 (95% CI 0.74 to 0.80) and an ICC of 0.87 (95% CI 0.85 to 0.89), showing substantial agreement between the ground truth and predicted B-line counts. **Conclusions**: ExoLungAI demonstrates a reliable performance in A-line detection and B-line detection/quantification. This automated tool has greater objectivity, consistency, and efficiency compared to manual methods. Many healthcare professionals including intensivists, radiologists, sonographers, medical trainers, and nurse practitioners can benefit from such a tool, as it assists the diagnostic capabilities of lung ultrasound and delivers rapid responses.

## 1. Introduction

Ultrasound imaging is a convenient, low-cost, non-invasive technology that works by sending sound waves through the body [1]. Those sound waves produce echoes when they pass from one tissue to another if those tissues have a different acoustic impedances [2]). In an aerated lung, the air rapidly dissipates the sound waves and, since there is no acoustic impedance mismatch, there is no echo that can be converted into an image. Indeed, in normal conditions, the only detectable structure is the pleura [3]. Therefore, it was thought for years that ultrasound would be of little use for the diagnosis of lung pathologies; however, it was later discovered that the artifacts visible in a lung ultrasound could be correlated to healthy and non-healthy lungs [4].

At present, lung ultrasound (LUS) serves an essential function in assessing pulmonary disorders at the point of care [5,6,7]. It has shown accuracy comparable to lung CT scans across a few key pathologies like these, and a higher sensitivity than chest X-rays for detecting pneumothorax and pleural effusion [8,9]. In particular, A-lines and B-lines represent two important sonographic indicators that provide essential insights into lung health [10].

A-lines are repeating horizontal echogenic lines that form parallel to the pleural line. The detection of A-lines indicates a high gas–volume ratio, so it generally suggests the presence of normal lung tissue, hyperinflation, or (in the absence of sliding lung) pneumothorax [5]. The presence of A-lines argues against pulmonary conditions such as pneumonia and pulmonary edema [11]. In contrast, B-lines are vertical echogenic lines that extend from the pleura to the end of the screen. Although the physical process that creates the B-lines is not entirely understood, their presence is associated with the presence of interstitial fluid or collagen tissue alterations [12,13,14].

The quantification of B-lines helps in the estimation of a disease’s severity, the monitoring of its progress, or the impact of a therapeutic intervention. For example, in some chronic diseases like heart failure, the clinician has to consider whether there is a change in lung fluid and revise their treatment approach accordingly [15]. While one or two isolated B-lines can be seen in healthy individuals [16,17], the presence of three to four B-lines per frame is correlated with a thickened lobular septa, and five or more B-lines indicate severe interstitial syndrome [5]. When two or more B-lines appear close together, they merge into a homogenous bright zone and form confluent B-lines. B-lines, particularly the confluent ones, indicate increased lung density and suggest a variety of pulmonary pathologies such as pulmonary edema, interstitial lung diseases, or acute respiratory distress syndrome (ARDS) [18]. In such cases, one method used to estimate the number of B-lines is to calculate the percentage of the scan occupied by B-lines, and then divide this percentage by ten [3].

Given the importance of assessing A-lines and B-lines, it is essential to have a reliable and objective approach to their detection. Previous studies have shown that the manual assessment of A/B-lines can be subjective [13]. Many factors like the clinician’s level of expertise, the quality of the images, the observer’s bias, and the complexity of this task can lead to high intra- and inter-observer variability [19]. Furthermore, manual interpretation and counting can be labor-intensive and time-consuming, requiring the clinician to review a video multiple times to make a decision. AI has emerged as a possible solution that improves objectivity and repeatability in this context. Additionally, AI tools can analyze a large volume of data in a fraction of a second and produce consistent results [12,20,21,22].

Another advantage of using AI is its ability to generate an initial automated report with preliminary findings for clinicians or radiologists, enhancing diagnostic efficiency [23]. This allows sonographers to rely on the tool’s findings, enabling them to concentrate more on the complex aspects of image acquisition. Medical trainers and educators can use such a system to teach new practitioners with the help of consistent examples [21,24]. Particularly in remote areas where access to experts is limited, an AI tool can provide insightful information for health practitioners so that they can refer a patient to an expert in case further examinations are needed [12,25]. However, all of these use cases are contingent upon the AI being reliable and trustworthy.

Having an explainable AI tool that highlights the features it uses for decision-making would enhance its trustworthiness [26]. A black box approach takes the LUS video and only outputs the number of A/B-lines present in it; although this approach can be accurate, it does not provide any insight into the intermediate steps that lead to the final decision. Alternatively, an explainable AI approach visualizes the A- and B-lines, displays the number of B-lines, and outputs the outcome according to the findings [27,28]. As a result, the user can see the basis for the outcome and trust the decision-making process in the latter system.

Here, we investigate the performance of ExoLungAI (Exo Imaging, Santa Clara, CA, USA, version 2.1.0), an AI algorithm used for analyzing lung ultrasound images. This AI tool is based on convolutional neural networks, which have demonstrated effectiveness in analyzing images and videos. Under the hood, the ExoLungAI utilizes a neural network capable of identifying A-lines and B-lines in LUSs. The ExoLungAI analyzes each frame in real time as the user scans, displaying the A-lines and B-lines in each frame in an online manner. The visualization reveals whether the B-lines are confluent or isolated. At the end of the scan, the tool determines the B-line count for the entire video by selecting the frame with the maximum number of B-lines. For the sake of B-line classification, this count is compared to a predetermined threshold (five in our case). If it exceeds the threshold, the scan is reported as a B-line case. In the case of A-lines, it counts the frames with A-lines. If this count exceeds a predetermined threshold, A-line presence is reported for the clip. The final classification of the presence of A-lines and B-lines helps the user determine the case’s severity.

### The Goals of This Investigation

In this paper, we aim to evaluate the performance of ExoLungAI, an AI tool designed to assess A-lines and B-lines in lung ultrasound scans. ExoLungAI identifies the presence or absence of A-lines and B-lines. If B-lines are present, the tool highlights and counts them and reports whether their number is greater than or equal to five. We compute the tool’s accuracy, specificity, and sensitivity by comparing its predictions (more than five B-lines) with the number of B-lines in the clinical report, similar to [29]. We further compare the number of B-lines predicted by the tool with the ground truth shown in the clinical report using the kappa score, intraclass correlation coefficient (ICC), and an error-distribution bar plot.

## 2. Materials and Methods

### 2.1. Study Design

We collected 692 scans from 48 patients who were admitted to the Emergency department (50%) or Intensive Care Unit (ICU) of the University of Alberta Hospital from April of 2021 to Jan of 2022 for COVID-19 symptoms. A total of 94% of the scans were obtained using a phased-array Philips Lumify, with the remaining acquired using a Clarius handheld probe. The study cohort had a mean age of 55 with a standard deviation of 12.39. The minimum age was 32 and the maximum was 77. A total of 35% of the participants in the study were female.

**Figure 1:**
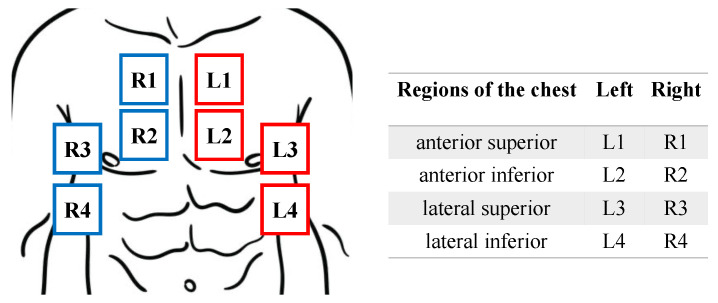
Illustration of lung regions divided into 8 zones.

Lung scans were taken following the same protocol described in [16,18,30]. Thus, for each patient, up to eight zones of the ventral wall of the chest, including the anterior superior, anterior inferior, lateral superior, and lateral inferior regions of both left and right sides, were collected, as illustrated in Figure 1. Lung scans were collected across days 1, 2, 3, 4, 5, and 7 unless the patient was discharged from the ICU or had an outcome of death before the 7th day.

The clinical report provides a video-level rough estimate of the percentage of lung area affected by B-lines, categorized as 100%, >75%, >50%, or normal. Based on these data and the ultrasound videos, two expert users annotated the exact video-level B-line grading scale from 0 to 10 (with 10 representing a 100% affected lung) and classified the absence or presence of A-lines. A consensus reading was reached through further discussion to obtain the final B-line count and A-line classification of the videos. There is a single A-line detection and B-line count label for each video. We followed [29] and categorized both the ground truth and predicted B-lines into two classes: less than 5 and 5 or more.

### 2.2. Approach

**Figure 2:**
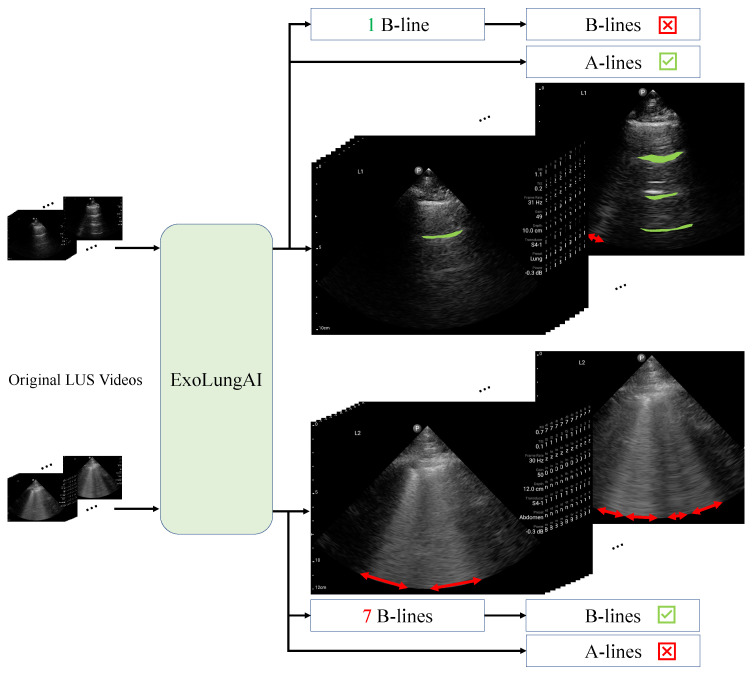
ExoLungAI workflow: visualization and classification of A-lines and B-lines from input lung ultrasound videos.

Figure 2 outlines the workflow of ExoLungAI for two example videos. First, we feed the original lung videos into the tool (left side of the figure). The tool visualizes the A-lines (in green) and B-lines (in red) for every frame of the video. Internally, the AI counts the number of B-lines based on what percentage of the rib space is affected by B-lines, a.k.a the instant percent method [30]. The frame with the maximum number of B-lines is selected and its count is reported as the clip’s B-line count. If the count is higher than a threshold of 5, it is reported as a B-line case. For the A-line classification of the clip, if the number of frames containing A-lines exceeds a threshold, the scan is reported as a case with A-lines. As seen at the top of the figure, the top video shows a healthy lung ultrasound with a clearly visible A-line and one B-line, which is fewer than five. This video is reported as a case with A-lines and no significant B-line involvement. In contrast, the video on the bottom shows 7 B-lines with no visible A-lines and is therefore reported as a B-line case.

The inference time for analyzing the A/B-lines in each frame is under 16 msec (∼60 frames per second). The AI tool can be seamlessly integrated into lung ultrasound workflows. As the physician is scanning different regions of the lung, they can observe the A-lines and B-lines in real time for each frame. The visualized A- and B-lines form the basis of the ultimate A- and B-line classification of the scan. The user can decide whether to accept or reject the final decision of the tool based on the clear and explainable real-time visualization provided.

### 2.3. Evaluation Metrics

This section describes the evaluation metrics used to assess the performance of ExoLungAI.

The B-line count represents the ratio of the rib space affected by B-lines, scaled by a factor of 10. This number ranges from 0 to 10, with 10 indicating that B-lines cover the entire pleura [30]. For B-line quantification, we calculated the error as the difference between the ground truth, as mentioned in the clinical report (GT), and the predicted B-line counts. The mean and standard deviation of errors across all cases was the metric used to determine on average how much this approach’s results deviate from the ground-truth B-line count. We also evaluated the agreement between the B-line count prediction and the ground truth in terms of Cohen’s kappa and intraclass correlation coefficients (ICCs).

For A- and B-line detection, we assessed the tool’s binary classification performance using accuracy, sensitivity, specificity, and Cohen’s kappa metrics. For B-line detection, we compared the results using two different thresholds: 3 and 5. We also presented the confusion matrix for both A- and B-line detection. A confusion matrix is a performance assessment tool that compares the predicted class labels (columns) to the true class labels (rows) and shows the distribution of these labels. The diagonal elements of the matrix represent the correctly classified cases.

We finally conducted a qualitative analysis to investigate some failure cases of ExoLungAI in both A-line detection and B-line detection/quantification. Since we care mostly about the sensitivity of this approach, we decided to present some of the cases in which the B-line count has the highest error (the GT is much greater than the predicted) and false positive cases of A-line detection.

## 3. Results

**Figure 3:**
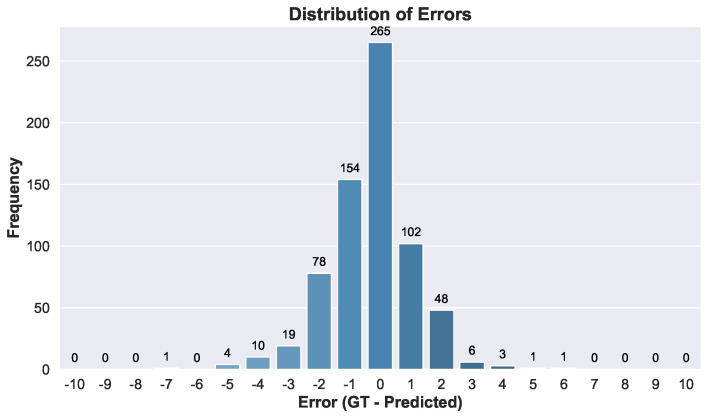
Distribution and frequency of errors across B-line classes.

We first compare our B-line counting results with the data from the clinical report. The bar plot in Figure 3 illustrates the distribution of errors in B-line counting across the videos. The mean error is −0.28, and the standard deviation is 1.4.

**Figure 4:**
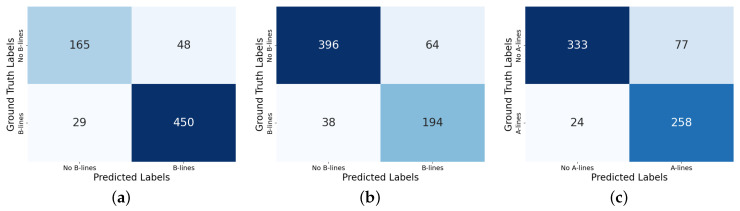
Confusion matrices for B-line and A-line detection. (**a**) B-line detection with threshold = 3. (**b**) B-line detection with threshold = 5. (**c**) A-line detection.

In Table 1, we compare the results of the A- and B-line binary classification of an AI tool with clinical ground-truth data. For B-line detection, we reported the results using two different thresholds, 5 and 3. When the final count is higher than the threshold we report it as a B-line case. The metrics that we use here for binary classification are true positives (TPs), true negatives (TNs), false positives (FPs), false negatives (FNs), accuracy, sensitivity, and specificity. Figure 4a–c show the confusion matrices for B-line detection at the thresholds 3 and 5, as well as that for A-line detection.

## 4. Discussion

Here we delve deeper into the results and examine their clinical relevance. As demonstrated in Figure 3, the presented tool gives favorable results, with most of the cases exhibiting zero or ±1 errors. The tool tends to slightly overestimate the count of B-lines in comparison to the clinical data. This is to ensure that the tool is sensitive and does not miss unhealthy cases, which is advantageous in clinical settings.

**Figure 5:**
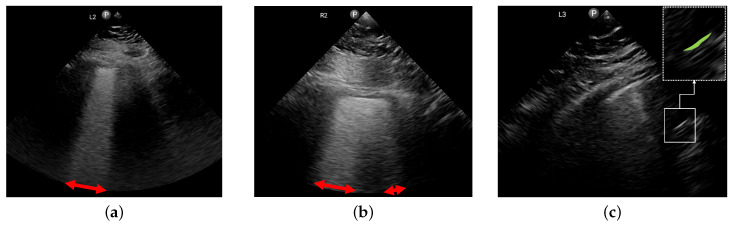
Some failure cases of the B- and A-line detection algorithm. (**a**) GT B-lines: 9; predicted B-lines: 3. (**b**) GT B-lines: 8; predicted B-lines: 4. (**c**) GT: no A-lines; tool predicted: A-lines.

We further qualitatively analyze some cases in which the error of the B-line prediction is high or an absence of A-lines is misclassified (see Figure 5).

Figure 5a shows a case in which the clinical report identified nine B-lines while the AI tool predicted three. As can be seen, although the tool correctly visualized the B-lines, it failed to calculate the proportion of the rib space affected by these B-lines. The same thing happened in Figure 5b (GT was eight and the prediction was four). The visualization of the B-lines is correct again but the method failed in its counting of the B-lines. In addition, based on the image itself, a lower count than eight could also be justified in this case. Note that the frame shown is the one with the largest affected area. For these cases, even though the final count of the B-lines may not be accurate, the explainability of the visualization remains valuable. The AI tool would still be able to assist the operator by clearly showing the affected parts of the lung field and enhancing their ability to identify these regions effectively.

Lastly, in Figure 5c we show a failure case of the A-line detection algorithm, where the poor quality of the image has impacted the tool’s performance, causing it to confuse an echogenic part of the image with an A-line, resulting in a false positive.

We evaluated the quantitative performance of the AI tool in detecting both A-lines and B-lines across several metrics in Table 1. As can be seen, both A-line and B-line detection methods show consistent performance, with a good balance across all metrics. The confusion matrices in Figure 4 show that most of the elements lie along the diagonal, indicating strong agreement between the algorithm’s predictions and the clinical ground truth for both A-line and B-line detection. As illustrated in the confusion matrices of Figure 4, ExoLungAI demonstrates a lower rate of false negatives compared to false positives in B-line classification (29/692 false negatives compared to 48/692 false positives). This signifies that the model prioritizes higher sensitivity, aiming to minimize missed true positive cases. The occurrence of false predictions could be due to lower image quality and/or the subjectivity of the ground truths (see, e.g., Figure 5a,b). For the case of A-line detection, false positives and false negatives formed 77 out of 692 and 24 out of 692 cases, respectively. Upon further investigation, we found that most false predictions were due to confusing image structures or artifacts resembling A-lines with actual A-lines in low-quality or suboptimal lung images (see, e.g., Figure 5c)

In the case of B-line detection, the user is free to change the B-line threshold to adjust the trade-off between sensitivity and specificity. If the operator needs higher sensitivity to spot B-line cases, they might decrease the threshold from 5 to 3 or pick any other threshold to obtain more true positives. However, at the same time, there would be a slight corresponding increase in false positives. It should be noted that the detection of A- and B-lines is highly subjective [13,30,31,32]. Many factors including image quality, the patient’s condition, and the proficiency of the user can be a source of inconsistency among observers. Thus, it is recommended that the user uses both the B- and A-line results to interpret the scan.

We explored the agreement between the B-line predictions and the ground truths in the context of the existing literature. The kappa score is a metric for quantifying the agreement between two raters. Anderson et al. investigated the B-line-counting inter-rater variability of six emergency physicians with experience in pleural sonography [30]. They classified scans based on whether they contained three or more B-lines. They reported a Cohen’s kappa score of 0.71. As mentioned in Table 1, we reached a kappa score of 0.73 for B-line classification with the same threshold, indicating a strong agreement between AI predictions and the clinical data. In the case of 10-class B-line counting, [32] reported a weighted kappa score of 0.67 (95% CI 0.50–0.84) and an ICC of 0.87 (95% CI 0.83 to 0.91) for the agreement between their automated B-line counter and the clinical data. Their ground-truth B-line counts’ mean and standard deviation were 1.60 and 1.35, respectively. Here we reach a weighted kappa score of 0.77 (95% CI 0.74 to 0.80) and an ICC of 0.87 (95% CI 0.85 to 0.89) for B-line counting on a dataset with a mean of 3.62 and standard deviation of 2.04, demonstrating a better agreement on a more diverse dataset. The narrower CIs in our ICC and kappa score indicate the higher reliability of the agreement in both metrics when using ExoLungAI.

## 5. Conclusions

A- and B-lines are instructive sonographic markers that help clinicians assess lung health at the point of care. In the absence of intervening air in the pleural space (i.e., pneumothorax), the presence of A-lines indicates a healthy aerated lung. On the other hand, a patient with numerous B-lines that are merged is likely to have a lung disease like pulmonary edema, interstitial fibrosis, or ARDS. Assessing the progression of these indicators over time helps the clinician diagnose the patient’s disease and tailor therapeutic interventions accordingly. Automating the detection of these findings could be helpful for three reasons: it introduces objectivity, improves inter-rater reliability, and enhances quality assurance through explainable AI. This has practical ramifications for the real-world use of ultrasound; access to and the learning of point-of-care ultrasound could become less of a barrier, while users in remote settings could receive real-time results when sonography experts are unavailable. Additionally, users can be more confident in their findings should there be a significant abnormality. Complementing the ultrasound device with an automatic lung-feature-detection tool that is consistent, reliable, and accurate can enhance users’ diagnostic capabilities in settings with fewer diagnostic means and expertise.

In this paper, we assessed the performance of our explainable-AI lung-sonographic-marker-detection tool, ExoLungAI. This tool takes a lung ultrasound video, visualizes the A-lines and B-lines of every frame in real time, and provides a final assessment by counting the B-lines and determining the presence of A-lines in the video, having been trained on 692 lung ultrasound POCUS videos. The results demonstrate that ExoLungAI achieved a competitive performance in A- and B-line detection. Specifically, the tool achieved a sensitivity of 84% and specificity of 86% for B-line detection and a sensitivity of 91% and specificity of 81% for A-line detection.

In conclusion, the automation of A-line detection and B-line detection/quantification using ExoLungAI has the potential to enhance lung clinical decision-making by providing a real-time visualization of A- and B-lines, as well as a retrospective binary classification of the presence of both markers. This automation allows clinicians to rapidly evaluate and determine the presence of pulmonary pathology without the need for expert overreads. We should note that despite the promising results of this AI tool, there are a few limitations here. First, the whole study was conducted in a single center (University of Alberta Hospital) and within a limited time frame, from April 2021 to January 2022. Secondly, this study only covers patients who were admitted for COVID-19 symptoms. Lastly, the focus of this study was solely on the evaluation of A-lines and B-lines for lung assessments. To achieve a more comprehensive understanding of the lung, it is essential to consider other lung features such as consolidation, pleural effusion, and lung sliding. Our future research will aim to enhance our method to evaluate these lung conditions, alongside A-lines and B-lines, using a larger dataset.

## Figures and Tables

**Table 1:** Summary of B-line and A-line performance metrics.

Metric	B-Line Threshold = 3	B-Line Threshold = 5	A-Lines
**Accuracy**	89%	85%	85%
**Sensitivity**	94%	84%	91%
**Specificity**	77%	86%	81%
**Kappa Score**	0.73 [95% CI 0.68–0.79], *p*-value < 0.05	0.68 [95% CI 0.62–0.74], *p*-value < 0.05	0.71 [95% CI 0.65–0.76], *p*-value < 0.05

## Data Availability

The dataset presented in this paper is not publicly available due to privacy concerns and the restrictions outlined in the data sharing agreement the authors have with the medical institution that obtained the data.
